# A Signal-On Fluorosensor Based on Quench-Release Principle for Sensitive Detection of Antibiotic Rapamycin

**DOI:** 10.3390/bios5020131

**Published:** 2015-03-26

**Authors:** Hee-Jin Jeong, Shuya Itayama, Hiroshi Ueda

**Affiliations:** 1 Chemical Resources Laboratory, Tokyo Institute of Technology, 4259-R1-18 Nagatsuta-cho, Midori-ku, Yokohama, 226-8503, Japan; E-Mail: heejin@pe.res.titech.ac.jp; 2 Department of Chemistry and Biotechnology, School of Engineering, The University of Tokyo, 7-3-1 Hongo, Bunkyo-ku, Tokyo 113-8656, Japan; E-Mail: itayama.0621@pe.res.titech.ac.jp

**Keywords:** fluorescent biosensor, fluorescence quenching, rapamycin, antibiotics, photoinduced electron transfer

## Abstract

An antibiotic rapamycin is one of the most commonly used immunosuppressive drugs, and also implicated for its anti-cancer activity. Hence, the determination of its blood level after organ transplantation or tumor treatment is of great concern in medicine. Although there are several rapamycin detection methods, many of them have limited sensitivity, and/or need complicated procedures and long assay time. As a novel fluorescent biosensor for rapamycin, here we propose “Q’-body”, which works on the fluorescence quench-release principle inspired by the antibody-based quenchbody (Q-body) technology. We constructed rapamycin Q’-bodies by linking the two interacting domains FKBP12 and FRB, whose association is triggered by rapamycin. The fusion proteins were each incorporated position-specifically with one of fluorescence dyes ATTO520, tetramethylrhodamine, or ATTO590 using a cell-free translation system. As a result, rapid rapamycin dose-dependent fluorescence increase derived of Q’-bodies was observed, especially for those with ATTO520 with a lowest detection limit of 0.65 nM, which indicates its utility as a novel fluorescent biosensor for rapamycin.

## 1. Introduction

Rapamycin (Sirolimus) is an antibiotic discovered from *Streptomyces hygroscopicus* in the soil of Easter island [1], and it is routinely used as a primary immunosuppressor for preventing kidney transplant rejection. Also, its anticancer [2] and life elongation [3] activities are suggested. Rapamycin exerts its various effects by its binding to FKBP12 (12-kDa FK506 binding protein) and then to FRB (FKBP12-rapamycin binding), and the formation of FKBP12-rapamycin-FRB ternary complex inhibits an mTOR (mammalian target of rapamycin), a Ser/Thr protein kinase that modulates signaling pathways regulating cellular growth and division [4,5,6]. Since the inhibition further prevents the regulation of the pathways related to human disorders such as tumor and other metabolic diseases including diabetes and obesity, there has been considerable demand for measuring the blood or organ concentration of administered rapamycin [2,7].

During the past decades, the development of biosensors has greatly improved its detection capability in a wide range of biological research and application areas [8,9]. Especially, protein-based fluorescent or bioluminescent biosensors, which combine the specificity provided by ligand-binding proteins and the sensitivity provided by the detection of their fluorescence and/or luminescence, has made an outstanding contribution in target identification including biomedical screening of drug compounds [10,11,12]. To date, several types of energy transfer-based optical sensors for the detection of rapamycin, based on Förster resonance energy transfer (FRET) and bioluminescence resonance energy transfer (BRET) have been reported [13,14]. As other rapamycin detection methods, protein-fragment complementation assay (PCA) assisted by split reporter proteins, such as β-lactamase [15], green fluorescent protein (GFP) variant [16] and firefly luciferase (Fluc) [17,18] are reported. However, all these methods use two proteins as a pair of probes comprised of two independent binding domains FKBP12 and FRB to probe rapamycin. Furthermore, the PCA-based probes have limitation of weak emission signal and limited thermal stability at their purified form.

As an alternative sensing approach, our group recently developed an innovative antibody-based reagentless fluorosensor Quenchbody (Q-body), which has been applied to the detection of a range of antigens including haptens, peptides as well as their modification, and larger proteins [19,20,21,22,23]. Typically, Q-bodies are constructed by incorporating a tethered organic fluorophore such as tetramethylrhodamine (TAMRA) to the N-terminal region of antibody fragment, where the fluorophore is quenched by photoinduced electron transfer (PET) from internal tryptophan (Trp) residues in antibody variable region (Fv). However, when antigen binds to antibody, the quenching of fluorophore is released by the antigen-induced structural change of Fv. Based on this principle, simple mixing of a Q-body with an antigen and subsequent fluorescence intensity measurement enables antigen quantitation in a simple and convenient manner. Thus, it has a great potential for biosensing applications requiring no additional reagents and washing steps.

**Figure 1:**
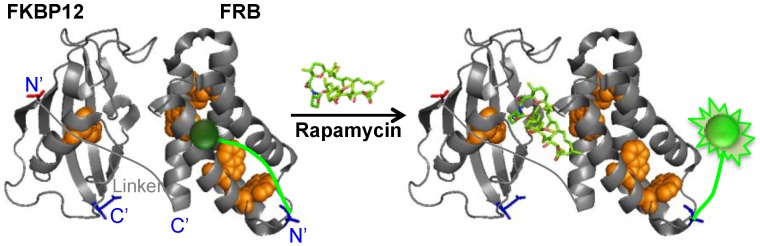
Scheme of rapamycin quenchbody (Q’-body) (based on protein data bank entry 1fap [6]).

Here, we aimed at developing a novel strategy for configuring a rapamycin biosensor by applying this quench-release principle. We constructed several fluorophore-protein conjugates named Q’-bodies, mimicking the Q-body concept, using an interacting protein pair FKBP12 and FRB whose interaction is mediated by rapamycin (Figure 1).

## 2. Experimental Section

### 2.1. Construction of Q’-Body Genes

The nucleotide sequences of primers used in this study (Operon-Eurofins, Tokyo, Japan) are summarized in Table 1. An FKBP12-FRB gene with N-terminal (G_3_S)_2_ and internal (G_4_S)_3_ linkers was prepared by splice overlap extension (SOE) PCR as follows. At first, synthetic human FKBP12 gene (Mr Gene GmbH, Regensburg, Germany) was amplified by PCR using primers FKBP-G3S2-Back and FKBP-linkFor. Also, synthetic human FKB gene (Mr Gene GmbH) was amplified by PCR using primers FRB-linkBack and FRB-For. Then the purified PCR products were mixed and amplified using primers G3S2-amb-Back and FRB-For to incorporate an amber codon at the N-terminal region. Finally, the product was inserted to *Nco*I- and *Sma*I-digested pROX-FL92.1amber plasmid [20] using an In-Fusion PCR cloning kit, resulting in *KR. To make *K*R gene, *KR gene was amplified by PCR using primers FKBP-G3S2-Back and Sal-link-amb-For, and the product was amplified using primers G3S2-amb-Back and Sal-link-amb-For. The amplified gene was inserted to *Nco*I- and *Sal*I-digested pROX-FL92.1amber as above. To make *RK, the *KR gene was made by SOE PCR using primer pairs FRB-G3S2-Back/FRB-linkFor, and FKBP-link-Back/FKBP-For, whose products were joined using primers G3S2-amb-Back and FKBP-For. The amplified gene was inserted to *Nco*I- and *Sma*I-digested pROX-FL92.1amber as above. To make *R*K, *KR gene was amplified by SOE PCR using primers FRB-G3S2-Back, FRB-linkFor, FKBP-For and FKBP-link-amb-Back, and then amplified using primers FKBP-For and G3S2-amb-Back. The amplified gene was inserted to *Nco*I- and *Sma*I-digested pROX-FL92.1amber. All amplifications were performed using KOD-plus-neo DNA polymerase (Toyobo, Osaka, Japan). The plasmids were prepared with PureYield plasmid miniprep system (Promega) and confirmed for the entire coding region sequences.

**Table 1:** Nucleotide sequences of primers used in this study.

Primer Name	Nucleotide Sequence (5'-3')
FKBP-G3S2-Back	GGTGGCGGTTCAGGTGGCGGTTCAATGGGCGTCCAGGTCGAG
FKBP-linkFor	GCTGCCACCTCCGCCTGAACCGCCTCCACCCTCCAGTTTCAGCAGCTCCAC
FRB-linkBack	GGCGGAGGTGGCAGCGGCGGTGGCGGGTCGACGCTCATTAGAGTGGCAATC
FRB-For	ATGAGAACCCCCCCCGAGCTGTTTTGAGATTCGTCT
G3S2-amb-Back	TAGTCTAATGAGACCGGTGGCGGTTCAGGTGGC
Sal-link-amb-For	CCACTCTAATGAGCGTCGACCCGCCACCGCCGCTGCCCTATCCGCCTGAACCGCC
FRB-G3S2-Back	GGTGGCGGTTCAGGTGGCGGTTCACTCATTAGAGTGGCAATC
FRB-linkFor	TCCGCCTGSSCCGCCTCCACCGAGCTGTTTTGAGATTCGTC
FKBP-link-Back	GGTGGCAGCGGCGGAGGTGGCAGCGGCGGTGGCGGGTCGACGATGGGCGTCCAGGTCGGAG
FKBP-For	ATGAGAACCCCCCCCCTCCAGTTTCAGCAGCTCCAC
FKBP-link-amb-Back	GGTGGCAGCGGCGGTTAGGGCAGCGGCGGTGGCGGGTCGACGATGGGCGTCCAGGTCGAG

### 2.2. Synthesis and Purification of Q’-Bodies

The synthesis of FKBP12-FRB fusion proteins position-specifically incorporated with fluorescence dye(s) was performed using an E. coli cell-free transcription-translation system using RYTS kit (Roche Diagnostics) with 10 mM oxidized glutathione, 75 ng of plasmid DNA, and 200 pmol of amber aminoacyl-tRNA conjugated with ATTO520, TAMRA, or ATTO590 (ProteinExpress, Chiba, Japan). The reaction mixture (25 μL) was incubated at 25 °C for 2 h and subsequently at 4 °C for 16 h.

To purify the synthesized full-length protein, a previously described protocol (Jeong *et al.*, 2013) was followed with minor modifications. First, a His Spin Trap Column (GE Healthcare) was primed by 500 μL of wash buffer (20 mM phosphate, 0.5 M sodium chloride (NaCl), 60 mM imidazole, 0.1% polyoxyethylene(23)lauryl ether, pH 7.4). The reaction mixture, which was diluted in 375 μL of wash buffer, was applied to the column. After incubation on a rotating wheel at 25 °C for 15 min, the column was washed three times with 500 μL of wash buffer. Finally, the Q’-body was eluted twice with 200 μL elution buffer (20 mM phosphate, 0.5 M NaCl, 0.5 M imidazole, 0.1% polyoxyethylene(23)lauryl ether, pH 7.4). The eluent was subjected to a Nanosep Centrifugal-3 k ultrafiltration device (Pall) and equilibrated with 500 μL of PBST (10 mM phosphate, 137 mM NaCl, 2.7 mM potassium chloride, 0.05% Tween 20, pH 7.4) two times to exchange buffers. The concentration of Q’-body was determined by comparing the fluorescence intensities of a known concentration of fluorescence dye and of the Q’-body under denaturing conditions in 7 M guanidium hydrochloride (GdnHCl) added with 100 mM dithiothreitol (DTT). All water used was purified using Milli-Q (Millipore, Tokyo, Japan). Other chemicals and reagents, unless otherwise indicated were from Wako pure chemicals (Osaka, Japan).

### 2.3. Fluorescence Measurements

Purified Q’-body (500 pM) in 250 μL of PBST containing 1% BSA was added in a 5 mm × 5 mm quartz cell (Jasco, Tokyo, Japan), and 1.25 μL of rapamycin (LKT Laboratories, St. Paul, MN, USA) in dimethylsulfoxide (DMSO) at variable concentration was added by titration. After each addition, the solution was incubated at 25 °C for two minutes prior to the spectral measurements. As a control, the same procedure was followed by adding DMSO instead of rapamycin solution. The fluorescence spectra were obtained at 25 °C using a fluorescence spectrophotometer Model FP-8500 (Jasco) with excitation at 524, 546, and 594 nm for ATTO520-, TAMRA-, and ATTO590-labeled Q’-body, respectively. The excitation and emission slit widths were set to 5.0 nm. Dose-response curves were fitted to a 4-parameter logistic equation at the maximum emission wavelength, using Kaleida Graph 4.1 (Synergy Software, Reading, PA, USA) and the EC_50_ value was calculated from the curve. The limit of detection (LOD) was calculated from the means and standard deviation (*n* = 20) of each dose and fitted dose-response curve according to the Armbruster’s definition [24].

## 3. Results and Discussion

**Figure 2:**
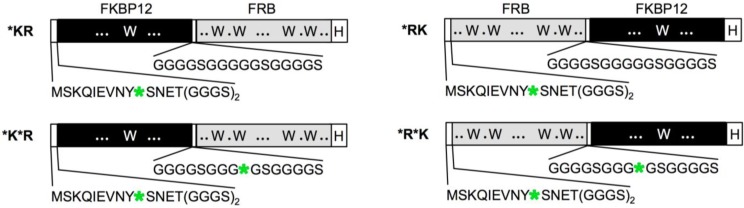
Schematic representation of Q’-body genes. Asterisk denotes an amber codon.

We constructed rapamycin Q’-body by tethering the FKBP12 and FRB with a flexible (G_4_S)_3_ linker, and incorporated fluorophore(s) to the specific site(s) of the fusion protein (Figure 1). We named this fluorescent protein as a “Q’-body” due to similarity in concept to Q-body, which is made of a single chain antibody (the two variable region fragments VH and VL of antibody tethered by a flexible linker) with site-specifically incorporated fluorophore(s). Since the position and number of the fluorophore might affect the fluorescent response, we constructed four different types of Q’-body genes, *KR, *K*R, *RK, and *R*K (Figure 2), wherein *KR is an FKBP12-FRB type gene with a 5'-terminal ProX-tag, which has an amber codon to introduce a dye (MSKQIEVNY*SNET, asterisk denotes an amber codon), and a 3'-terminal His-tag. The sequence of *K*R is the same as *KR, except for an additional amber codon introduced in the middle of the interdomain linker. Considering the fact that more Trp residues exist in FRB (four) than FKBP12 (one), constructs with reversed domain order *RK and *R*K were made similarly. To investigate the effect of dyes, we used three rhodamine derivatives to examine their performance as a suitable label for Q’-body. In addition to TAMRA and ATTO520, which are proven good labels for a Q-body, a new fluorophore ATTO590 with longer excitation/emission wavelengths with possible *in vivo* applications was examined.

**Figure 3:**
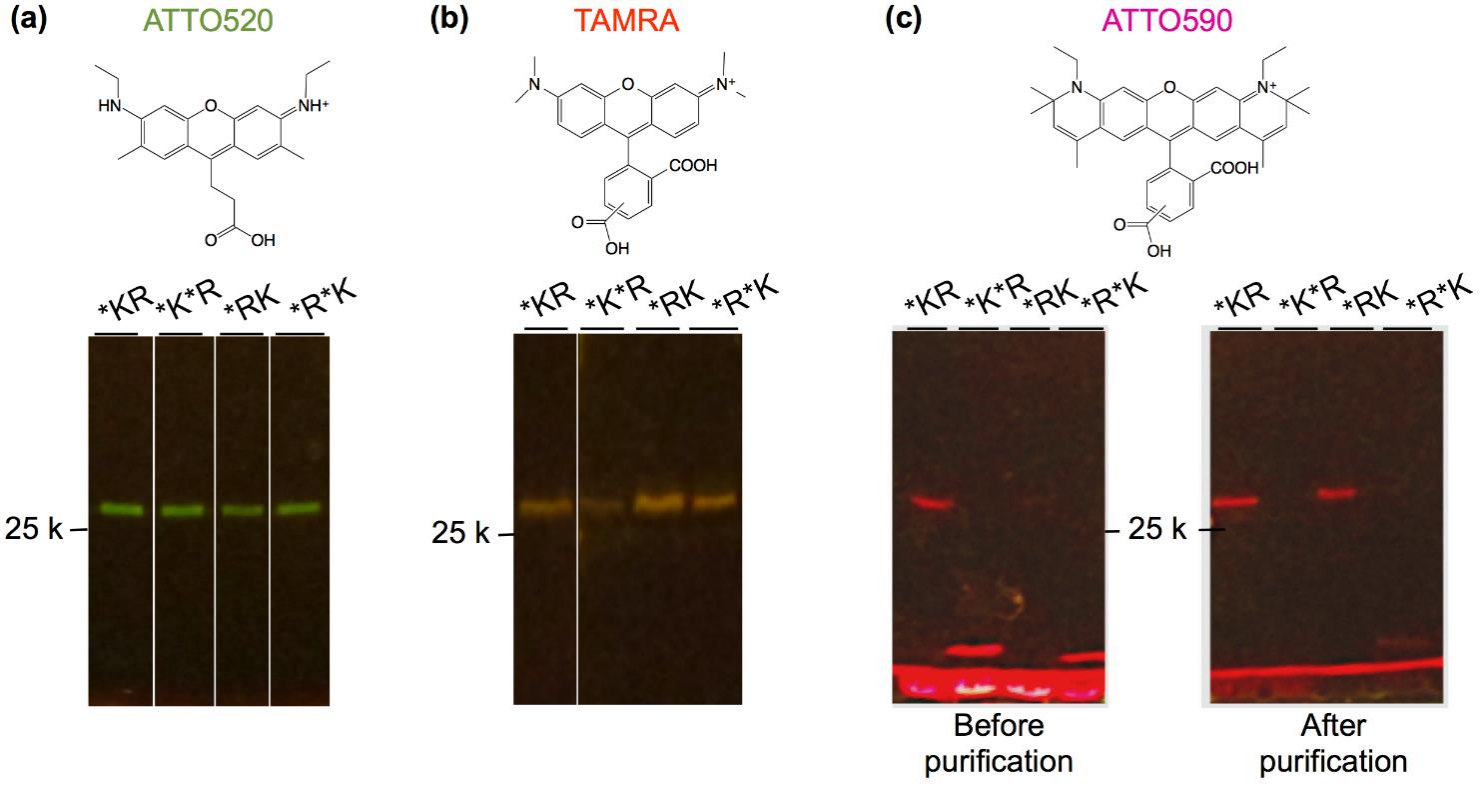
Fluorescence image of (**a**) ATTO520-; (**b**) tetramethylrhodamine (TAMRA)-; and (**c**) ATTO590-incorporated Q’-bodies. Scheme above each gel is the chemical structure of the dye used.

Twelve kinds of Q’-bodies with different configurations and fluorophores were synthesized and purified by immobilized metal affinity chromatography, and the fluorescence images of the SDS-PAGE gels were taken (Figure 3). Clear bands of ATTO520- and TAMRA- labeled Q’-bodies were observed at above the 25 kD marker, indicating successful expression of these proteins with incorporated fluorophores. On the other hand, in the case of purified ATTO590-labeled Q’-bodies with two dyes, faint bands of full-length proteins were observed. When non-purified proteins were applied to the gel, a prominent band at smaller molecular weight of ~15 kD was observed in the cases of K*R* and R*K*. In this study, we used aminoacylated amber suppressor tRNAs to introduce unnatural amino acids [25]. Since the amber codon is a stop codon, the synthesis of double-labeled full-length proteins would be less efficient due to imperfect suppression efficiency. As a result, the full-length protein was mostly removed after purification via the C-terminal His_6_-tag. In particular, the larger size of ATTO590 than other two (molecular weights of ATTO590, TAMRA and ATTO520 are 691.2, 430.5 and 466.9, respectively) and its linear and consecutive five benzene rings might be unfavorable for the efficient incorporation. Nevertheless, the purified proteins with single ATTO590 dye were mostly full-length.

**Figure 4:**
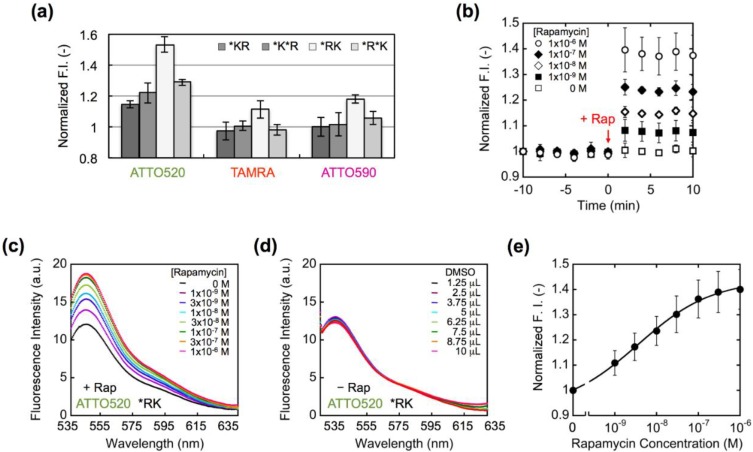
(**a**) Comparison of the fluorescence responses of Q’-body upon addition of 1 μM rapamycin. Error bars represent ±1 SD (*n* = 3). (**b**) Fluorescence time-course of ATTO520-incorporated *RK type Q’-body after adding rapamycin. (**c**) Fluorescence spectra of ATTO520-incorporated *RK type Q’-body in the presence of rapamycin at indicated concentrations. (**d**) The same as in (c) except that DMSO, the solvent of rapamycin, was added. (**e**) Rapamycin concentration-dependent fluorescence increase of ATTO520-incorporated *RK type Q’-body Error bars represent ±1 SD (*n* = 20).

We measured the fluorescence intensity of purified Q’-body in the absence and presence of 1 µM rapamycin (Figure 4a). As a result, the fluorescence intensity was increased within 2 minutes after adding rapamycin (Figure 4b). However, the obtained responses were different from each other depending on the configuration of Q’-body and the dye used. Among the four constructs, *RK-type Q’-bodies showed the highest rapamycin-dependent fluorescence enhancement, regardless of the fluorophores. Since there are four Trp residues in FRB and one in FKBP12, it is probable that the fluorophore has more chances to be quenched when it is placed closer to FRB. Unexpectedly, unlike our previous Q-bodies [21], double-labeled Q’-bodies showed lower responses than the single-labeled ones. Among the three dyes used, ATTO520-labeled Q’-bodies showed the highest response for all the types of constructs. This difference most likely reflects that of fluorophore’s chemical structures. ATTO520 is less hydrophobic and smaller in size, and has a positive charge unlike TAMRA and ATTO590, which might result in its lower tendency to dimer-induced quenching (H-dimer formation) than TAMRA and ATTO590. Possibly, the interface between FKBP12 and FRB is less hydrophobic than that of V_H_ and V_L_, thus the less hydrophobic dye was more prone to enter the interface and more efficiently quenched. Also, in the cases of TAMRA and ATTO590, intermolecular dye-dye interaction might have made these dyes quenched irrespective of rapamycin, thus might have prevented their efficient responses. Alternatively, as the molecular size of ATTO520 is smaller than other dyes and the distance between the dye and protein would be shorter, the dye might be more effectively quenched by Trp residues [26].

Meanwhile, since the interaction between FKBP12 and FRB proceeds via the fast binding of rapamycin to FKBP12, which is followed by the binding of FRB to this complex [4], it is theoretically possible that the two Q’-bodies with rapamycin-bound FKBP interact each other to form a dimer, like scFv dimer or diabody. However, in the case of scFv-based Q-body in our previous report [20], although the existence of such dimer is implied from single molecule fluorescence measurement, it did not seriously affect the Q-body’s fluorescent behavior. This was probably due to negligible structural difference between monomer and dimer in terms of local structure around the dye. At this moment we think this is also the case for Q’-body. However, more detailed analysis of the effect of multimers is an interesting future project. Using ATTO520-labeled *RK type Q’-body, which showed the highest response among the twelve types, the rapamycin dose-dependency of fluorescent spectra was measured, as well as control experiment adding equal volumes of DMSO (Figure 4c,d). The resultant normalized dose-response is shown as Figure 4e.

From Figure 4e, the EC_50_ and LOD values of ATTO520-labeled *RK type Q’-body were calculated as 3.6 nM and 0.65 nM, respectively. These values are sufficiently low to allow the detection of rapamycin concentration in allografted rat [27] and in human blood at therapeutic settings [28,29]. This high sensitivity is probably due to the linkage of the two proteins, and is comparable or higher than the reported sensitivity obtained by other assays [16,30], indicating its potential utility as a diagnostic reagent.

**Figure A1:**
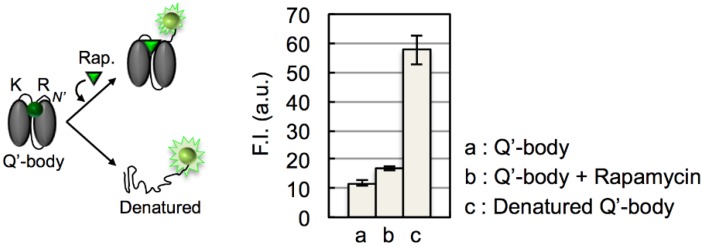
Schematic representation of quenched, de-quenched and denatured Q’-body (**left**); and the maximum fluorescence intensity for each Q’-body (**right**). In b and c, 1 µM rapamycin and 7 M GdnHCl/100 mM DTT were added, respectively. Error bars represent ±1 SD (*n* = 3).

When we performed denaturation-induced de-quenching of ATTO520-labeled *RK Q’-body with 7 M GdnHCl and 100 mM DTT, the fluorescence intensity increased to ~4.9-fold (Figure A1). In other words, the dye is first quenched to ~20%, and possible maximum de-quenching of this Q’-body will be ~4.9-fold. However, the currently observed rapamycin-dependent de-quenching was modest 1.5-fold. Hence, to attain higher fluorescent response, the improvement of rapamycin-induced de-quenching efficiency will be the most effective strategy.

## 4. Conclusions

We report a sensitive signal-on rapamycin sensor utilizing quench-release phenomenon of the fluorescent dye incorporated to the tip of a binding protein pair. Compared with other rapamycin detection methods, the developed sensor is remarkably simple and does not require any reagents and washing steps to perform the assay. This sensor will be further applied to the imaging of rapamycin distribution *in situ* or *in vivo*, although some increase in response might be necessary. This will be possible by more extensive adjustment of dye position to attain higher de-quenching, as in the case of Q-body [22]. In future, the Q’-body principle will be exploited with many other binding proteins for the detection of various drugs and biomarkers. We believe that this study represents a step forward to the application of quench-release principle to non-antibody binding scaffolds.
